# An Acceleration-Based Gait Assessment Method for Children with Cerebral Palsy

**DOI:** 10.3390/s17051002

**Published:** 2017-05-02

**Authors:** Xiang Chen, Songmei Liao, Shuai Cao, De Wu, Xu Zhang

**Affiliations:** 1 Department of Electronic Science and Technology, University of Science and Technology of China, Hefei 230026, China; lsmnynl@mail.ustc.edu.cn (S.L.); caoshuai@ustc.edu.cn (S.C.); xuzhang90@ustc.edu.cn (X.Z.); 2 Department of Pediatrics, The First Affiliated Hospital of Anhui Medical University, Hefei 230026, China; wude7310@sohu.com

**Keywords:** acceleration, cerebral palsy (CP), gait assessment, grey relational analysis

## Abstract

With the aim of providing an objective tool for motion disability assessment in clinical diagnosis and rehabilitation therapy of cerebral palsy (CP) patients, an acceleration-based gait assessment method was proposed in this paper. To capture gait information, three inertial measurement units (IMUs) were placed on the lower trunk and thigh, respectively. By comparing differences in the gait acceleration modes between children with CP and healthy subjects, an assessment method based on grey relational analysis and five gait parameters, including Pearson coefficient, variance ratio, the number of extreme points, harmonic ratio and symmetry was established. Twenty-two children with cerebral palsy (7.49 ± 2.86 years old), fourteen healthy adults (24.2 ± 1.55 years old) and ten healthy children (7.03 ± 1.49 years old) participated in the gait data acquisition experiment. The results demonstrated that, compared to healthy subjects, the symptoms and severity of motor dysfunction of CP children could result in abnormality of the gait acceleration modes, and the proposed assessment method was able to effectively evaluate the degree gait abnormality in CP children.

## 1. Introduction

Cerebral palsy (CP) was a syndrome which refers to a group of permanent disorders of movement and posture, and caused by abnormal development of, or damage to the brain in the developing fetal or infant [1,2,3]. Patients suffering from CP are often associated with upper and lower extremity motor dysfunction, where their lower limb movement disorder often leads to various forms of abnormal gait [4]. Compared with children with typical development, the gait of children with CP is usually asymmetric, slow and less stable [1]. Gait rehabilitation training is important for the walking ability recovery of CP patients, and it is of great significance to do gait analysis of CP during the gait rehabilitation process. 

Clinically, abnormal movements of patients are usually analyzed visually or using questionnaires. For example, the Ashworth Scale, Modified Ashworth Scale and Tardieu Scale are usually used to assess the spasticity of patients by measuring the level of resistance to passive movement [5,6]. The movement dysfunction of patients with CP is usually assessed by the gross motor function classification system (GMFCS) and gross motor function measure (GMFM, including GMFM-88 and GMFM-66) based on self-initiated movements, with emphasis on sitting, transfers, and mobility [7,8,9]. GMFCS was defined as a five-level classification system, in which the distinctions between levels were determined by the quality of movement or the need for hand-held mobility devices [7,8]. For GMFM-88, activities in lying and rolling up to walking, running and jumping skills were included in the 88 items, and for each item, there was a 4-point scoring system [9]. GMFM-66 comprises a subset of GMFM-88, and is only used to measure the gross motor function of children with cerebral palsy [9]. For both GMFCS and GMFM, one drawback is that they are related to the experience of clinicians and may produce subjective results. What’s more, for GMFM, it can take a long time to complete an assessment, even for someone familiar with the measures [10]. Therefore, it is meaningful to develop an approach to evaluate motor function of CP patients in an objective and convenient way. 

Gait analysis had attracted a considerable amount of research interest since the late 19th century [11]. Most gait analytical methods have used a variety of sensor technologies to capture kinematic and kinetic information during walking [12,13,14,15,16]. Camera-based motion capturing systems combined with motion sensors or force platforms are usually used in related studies [13,14]. Although these systems can capture accurately kinematic and kinetic parameters of the hip, knee, ankle etc., they are often very expensive and only suitable for use in laboratory settings. On the other hand, small, low-cost and wearable sensors like accelerometers and gyroscopes, inertial measurement units (IMUs) and others have also been efficiently used in gait analysis [12,15,16,17,18,19,20]. IMUs can measure the acceleration and angular velocity data of body segments they are attached to, which is usually used to estimate joint kinematics or centre of mass (COM) movement and so on. In many studies, acceleration signals have demonstrated good performance in gait measurement [21,22,23,24,25,26]. Zijlstra et al. [21] found that the acceleration patterns of the waist in healthy adults were consistent with the different moments during a gait cycle, and could be predicted well by an inverted pendulum model of the body’s COM trajectory [23]. Bugané et al. analyzed the acceleration of the waist in depth and discussed the relationship between acceleration signals and gait parameters [24]. In Huisinga et al.’s study [25], the Lyapunov exponent in the medio-lateral (ML) and anterior–posterior (AP) direction, and root mean square (RMS) in the AP direction of trunk acceleration were found to display significant differences between persons with multiple sclerosis (PwMS) and healthy controls. In the study of Mizuike et al., the raw RMS and autocorrelation of trunk acceleration were found to show significant differences between stroke patients and healthy subjects [26]. In the meanwhile, there were also some studies that used acceleration to investigate the motion abilities of CP children [27,28]. In the study of Iosa et al., gait stability and symmetry were explored by means of trunk acceleration signals, and the RMS, harmonic ratio (HR), the minimum peaks of accelerations, the ratio index of minimum peaks and so on were analyzed, and significance differences in these parameters were found between CP children and healthy children [27]. In Saether et al.’s study, the asymmetry and RMS of trunk acceleration were found to show significant differences between the gait of healthy children and CP children [28]. The similarities between all the above studies was their intent to explore the differences in gait characteristics between children with cerebral palsy and healthy children, but quantitative assessments of abnormal gait in children with cerebral palsy were not involved. 

To meet the need for an objective gait analysis for CP sufferers in clinical diagnosis and rehabilitation treatment, the novelty of this study is that a gait assessment model based on the characteristics of gait acceleration signals was proposed for the quantitative assessment of the gait abnormalities of CP patients. Five gait parameters, including Pearson coefficient [29], which describesd the similarity between abnormal gait of CP children and standard gait patterns in healthy adults, variance ratio [30], which measures the repeatability over gait cycles, the number of extreme points [31], which is one reflection of stability, harmonic ratio [32], which shows the smoothness and rhythm of gait patterns and symmetry [33], which shows the step regularity, were selected as gait parameters. The gait assessment model was established based on grey relational analysis [34,35].

## 2. Materials and Methods 

The main framework of this study consisted of three major steps: firstly, an IMU-based gait data acquisition and pre-processing framework were designed; then gait acceleration (ACC) data from healthy adults were analyzed to extract representative Characteristic Gait Graphs (CGGs); finally, an ACC-based gait assessment model was proposed based on the gait parameters and grey relational analysis.

### 2.1. Subjects

Fourteen healthy adults (AD group, 24.2 ± 1.55 years old, height: 169.7 ± 6.23 cm, weight: 58.78 ± 9.34 kg), ten healthy children (TD group, 7.03 ± 1.49 years old, height: 125.6 ± 11.86 cm, weight: 24.4 ± 6.05 kg) and 22 children with CP (CP group, 7.51 ± 2.96 years, height: 117.91 ± 25.53 cm, weight: 26.14 ± 6.23 kg) participated in the data collection experiments. All CP children were recruited according to their GMFCS level with the help of a clinician. The inclusion criteria for CP children included: (a) being clinically diagnosed with cerebral palsy; (b) being classified as level I, II and III of the Gross Motor Function Classification System; (c) having the ability to walk independently or walk with assistance; (d) having no history of surgery [36]. Informed consent forms were signed by the children’s guardians before the experiments and the study was approved by the Ethics Review Committee of Anhui Medical University (No. PJ 2014-08-04). Detailed information about the CP subjects is listed in Table 1. 

### 2.2. Data Acquisition and Preprocessing

#### 2.2.1. Data Acquisition

**Figure 1:**
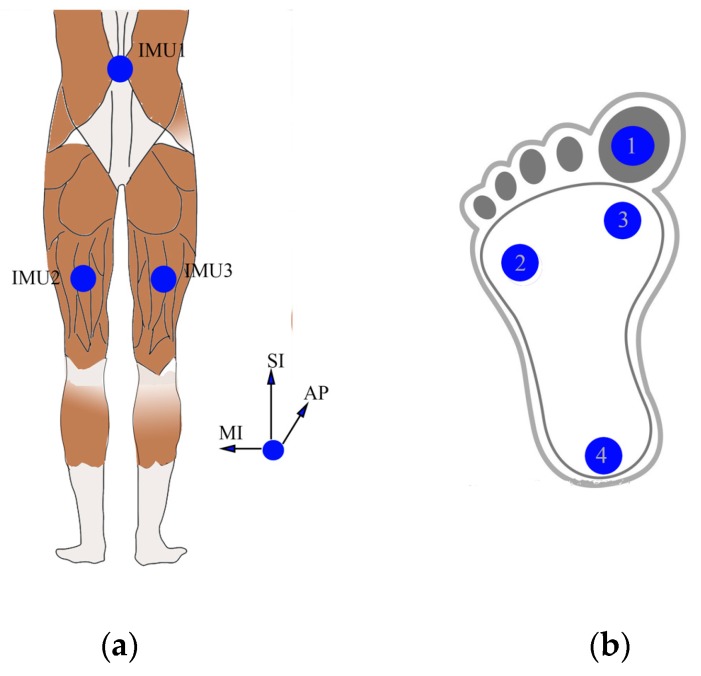
Locations of: (**a**) IMUs, AP, SI and ML represent the anterior–posterior, superior–inferior, medio-lateral directions respectively; (**b**) FSRs (1: toe, 2: fifth metatarsophalangeal, 3: first metatarsophalangeal, and 4: heel).

Three IMUs (MPU9250, InvenSense, San Jose, CA, USA) including a 3-axis ACC and a 3-axis GYRO were used to capture gait data in this study. All IMUs were checked to establish the offset values for acceleration and angular velocity data [37]. Under the help of a clinician, IMU1 was mounted on subjects’ back area corresponding to the L2–L3 spinous process, which is close to the body center of mass, and the other two were mounted on the middle of the right and left thigh semitendinosus, respectively as Figure 1a shows. In addition, an operation to align sensible axes of IMU with the sagittal, coronal, transverse axes of each body segment, was done to ensure the alignment precision [38,39]. All IMUs were attached by muscle stickers. For subjects AD9, TD5, CP8, CP12 and CP22, four force sensors (Interlink Electronics FSR402，Camarillo, CA, USA) were also placed under the toe, first metatarsal head, fifth metatarsal head and heel respectively as Figure 1b shows, and the force data were mainly used during the preliminary experimental session to verify the effectiveness of the gait segmentation algorithm based on peak detection of AP acceleration.

Before the data collection experiments, all subjects were given 3 min to get accustomed to the experimental procedure and were instructed to stand still for about 5 s before walking. Then, ten healthy adults and six healthy children and all CP children were instructed to walk for 30 consecutive strides at their self-selected speed. To further explore the influence of walking speed on the proposed gait assessment model, the other four healthy adults and four healthy children were asked to walk for 30 consecutive strides at slow, self-selected, fast speed respectively. All signals were sampled at 100 HZ and data were saved to disk for off-line analysis using Matlab 7.14 (Mathworks Inc., Natick, MA, USA). 

#### 2.2.2. Pre-Processing 

Since the orientations of acceleration changed during walking, a quaternion-based method was used to transform the acceleration to a common reference frame with axes in anterior–posterior (AP), superior–inferior (SI) and medio-lateral (ML) directions [18,40,41]. The specific process could be described as follows: firstly, the initial sensor orientation and initial quaternions were calculated using the 5 s static standing data. Then, the angular velocity from the gyro sensor was used to update quaternions [18,41]. Finally, the acceleration was transformed to a global system by matrix multiplication of raw acceleration and the quaternion matrix. In the above process, the initial offset values of the sensors were used for converting measured values to actual acceleration and actual angular velocity during the analysis [39]. According to the study of Karantonis et al. [42], the frequency components of most body movements are below 20 Hz, so a low-pass filtering step (window-based finite impulse response filter, 50th order, cutoff of 20 Hz) was applied to reduce the high frequency noise of the angular velocity and acceleration signals in this study.

#### 2.2.3. Gait Segmentation

**Figure 2:**
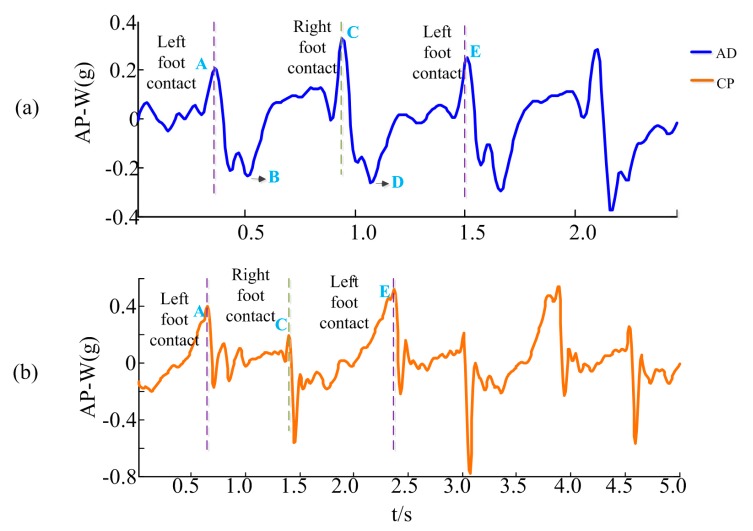
Gait acceleration from (**a**) a healthy subject; (**b**) a CP child.

To obtain gait acceleration data from continuous signals, gait segmentation was performed before gait analysis. Some methods like zero crossing detection of the normalized acceleration, maxima and minima detection of vertical acceleration and peak detection of AP acceleration (all were trunk acceleration) have been introduced for gait segmentation in related studies [22,43]. Based upon an inverted pendulum model of the body’s centre of mass trajectory [23], the peak detection of AP acceleration method proposed by Zijlstra and HoF has been used widely [22]. As shown in Figure 2a, which gives an example of AP waist acceleration (AP-W) of typical gait cycles from a healthy subject, a positive peak (point A) can be observed in the AP-W acceleration signal when the left heel touches the ground. When the entire left foot touches the ground, the acceleration signal decreases rapidly, and a valley point (point B) appears. The signal increases when the right foot starts moving forward. When the right heel contacts the ground, a peak (point C) also appears, and the process from C to E is similar to the process from A to C. For CP children, the peaks corresponding to foot touchdowns still exist in the AP-W signals, as shown in Figure 2b. Accordingly, positive peak points including A and C points could be regarded as the start point of a gait cycle. In this study, the positive peak points of AP direction of waist acceleration were detected by a window peak detection algorithm, in which the window was an estimation of the step length of each subject [44]. After gait segmentation, several gait cycles of start and end moments were removed. In addition, the gait cycles whose lengths larger than twice of or less than one half of the average length of the remaining gait cycles were also removed.

### 2.3. Representative Gait Acceleration Signals, Average Gait Graph (AGG), and Characteristic Gait Graph (CGG)

Gait acceleration pattern analysis was conducted on 3 × 3 channels acceleration data corresponding to three directions and three positions collected from healthy adults firstly. The goal of gait acceleration pattern analysis was to find which of these nine channels can describe the gait of healthy adults stably, independently of subject’s age or gender. To make the gait acceleration signal comparable among subjects, the length of each gait cycle was firstly adjusted to a fixed value *M* (*M* = 100 points) by linear interpolation or downsampling [45]. Then, for each subject, the average of three normalized gait cycles was calculated. Finally, the correlation coefficients of these average gaits between subjects were calculated and the channels which obtained high similarity (*r* ≥ 0.75) among healthy adults were selected as representative acceleration channels for gait acceleration pattern extraction. 

After representative acceleration channels were determined, average gait graphs (AGGs), which were defined to represent each person’s average gait, were obtained by calculating the average of the representative accelerations of three gait cycles. In this study, four AGG were extracted for each subject and used in the further gait assessment model. AGGs were found to be similar among healthy subjects. For CP children, AGGs could reflect their gait abnormalities during walking. Usually the more severe the patients’ condition was, more noise and glitches existed in their AGGs. Because similar gait acceleration patterns existed in healthy adults, a characteristic gait graph (CGG), which was defined as the average of AGGs of several healthy adults, was defined as the standard gait acceleration pattern for further gait assessment.

### 2.4. Gray Relational Analysis Based Gait Assessment Model

#### 2.4.1. Gait Acceleration Parameters

**Figure 3:**
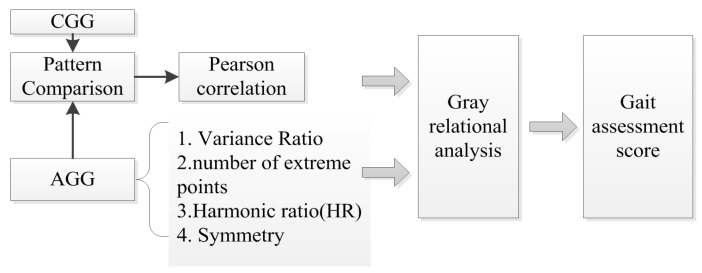
The flow chart of our gait assessment model.

A gait assessment framework was proposed to evaluate the gait abnormality of children with CP scientifically, as shown in Figure 3. 

**Figure 4:**
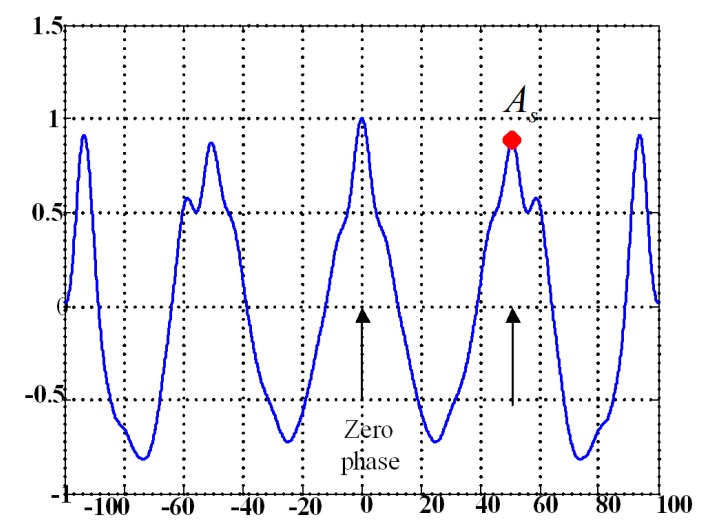
An example of an unbiased autocorrelation sequence of AP-W acceleration signals during walking.

In this study, five gait features including Pearson coefficient, variance ratio, the number of extreme points, harmonic ratio and symmetry, were used to comprehensively describe the acceleration signal and evaluate the abnormal degree of gait:

- Pearson coefficient: The Pearson coefficient reflects the degree of linear correlation between two variables, where a large value indicates a strong correlation [29]. In this study, *r* was calculated by Equation (1) to represent the degree of similarity of the corresponding channel in the AGG and CGG:
  (1) $$r=\frac{1}{M-1}\sum_{i=1}^{M}(\frac{X_{i}-\bar{X}}{S_{X}})\left(\frac{Y_{i}-\bar{Y}}{S_{Y}}\right)$$
  $X_{i}$ and $Y_{i}$ represent the values of each channel in the AGG and CGG, respectively, $\bar{X}$, $S_{X}$, $\bar{Y}$, $S_{Y}$, represent the mean and standard deviation of each channel in the AGG and CGG, and *M* is the length. Then P, which represents the overall correlation, is defined as the sum of the *r* values of all channels. A high P means that the AGG is similar to the CGG, and its maximum value is *K.* Here *K* is the number of the representative acceleration channels.
- Variance Ratio (VR): Variance ratio, proposed by Hershler and Milner, was used to measure the repeatability of a signal over a given number of identical footsteps [30]. In this study, the repeatability of the three gait cycles, which was used to obtain the AGG, was calculated. In Equation (2), *M* is the fixed length, *n* is the number of gait cycles, $X_{ij}$ is the value of the *j*th gait cycle at time *i*, $\bar{X_{i}}$ is the average of the three gait cycles values at time *i*, and $\bar{X}$ is the grand mean of the average gait signal. If the values of the corresponding channels are similar between three gait cycles, *v*→0. Finally the parameter V is defined as the sum of *v* in all channels to depict the repeatability of the three gait cycles:
  (2) $$v=\frac{\sum_{i=1}^{M}\sum_{j=1}^{n}{\left(X_{ij}-\bar{X_{i}}\right)}^{2}/k\left(n-1\right)}{\sum_{i=1}^{M}\sum_{j=1}^{n}{\left(X_{ij}-\bar{X}\right)}^{2}/\left(kn-1\right)}$$
- The number of extreme points: During walking, children with CP often swung accompanied by some involuntary shaking, and this could result in unstable gait acceleration with more burr and extreme points, which was used to evaluate the upper limb function in Yanran et al.’s study [31]. As we know, the extreme points can be defined as the point whose first left and right side derivatives have a different sign. By finding two adjacent values which have opposite symbols in the first derivative of AGG, extreme points could be obtained. N, defined as the sum of the extreme points in all representative channels, is used to represent the extreme point feature in this study.
- Harmonic ratio (HR): The harmonic ratio is used to provide an indication of the smoothness and rhythm of acceleration patterns, and it is based on the premise that the unit of measurement from a continuous acceleration signal is a stride and the acceleration pattern repeated twice [32]. Hence, only the acceleration of the waist was analyzed in this study. Using a finite Fourier series, the acceleration signal could be considered as the combination of even and odd harmonics, and the harmonic ratio h could be calculated using Equation (3). In this study, the sum of two *h* values obtained from the SI-W and AP-W in AGGs, respectively, was used to represent the overall harmonic ratio, represented by H:
  (3) $$h=\frac{\sum_{n=2,4,6,\ldots }^{20}C_{n}}{\sum_{n=1,3,9,\ldots }^{19}C_{n}},$$
- Symmetry: Normal human walking is composed of cyclical movements of the lower limbs, and the movement of the left and right legs should be symmetrical. According to the study of Moe-Nilssen [33], unbiased autocorrelation coefficients of gait waist acceleration represent a periodic sequence. As shown in Figure 4, which demonstrates an unbiased and normalized autocorrelation sequence of the waist acceleration in the AP axis, the value of point $A_{s}$ could express the regularity of the acceleration signal between neighboring steps, and a higher $A_{s}$ means a better step regularity [33]. In this study, the sum of two $A_{s}$ values obtained from the autocorrelation coefficients of SI-W and AP-W in AGGs, respectively, were used to represent gait symmetry, represented by S.

#### 2.4.2. Gait Assessment Method Based on Grey Relational Analysis

The basic idea of the grey relational analysis method [34] is to determine the relevance between a comparison set and a reference set according to the degree of similarity of their geometrical curves. Given a comparison set X(m×n) and a reference set R(1×n), grey relational analysis usually consists of the following three steps:

*Step 1*: The normalized comparison set Z(m×n) is obtained by standardizing the comparison set X(m×n);
*Step 2*: The correlation coefficient $\xi_{i}\left(j\right)$ between the *i*th row of *Z* and *R* is computed using Equation (4). The distinguishing coefficient γ is between 0 and 1, and was set as 1 in this study:
  (4) $$\xi_{i}\left(j\right)=\frac{minAbs+\gamma \times maxAbs}{\left|Z_{i}\left(j\right)-R\left(j\right)\right|+\gamma \times maxAbs}\;i=1,2,\ldots m;j=1,2,\ldots n$$
  (5) $$\mathrm{where}\{\begin{array}{c} minAbs=\underset{1\leq i\leq m}{\min}\left\{\underset{1\leq j\leq n}{\min}\left(\left|Z_{i}\left(j\right)-R\left(j\right)\right|\right)\right\}\\ maxAbs=\underset{1\leq i\leq m}{\max}\left\{\underset{1\leq j\leq n}{\max}\left(\left|Z_{i}\left(j\right)-R\left(j\right)\right|\right)\right\} \end{array}$$
*Step 3*: The correlation degree $C_{i}$ between the $i^{th}$ vector of *Z* and the reference set *R* with weights $\omega_{j}$ and $\xi_{i}\left(j\right)$ is calculated by Equation (6). $C_{i}$ was between 0 and 1, and a higher $C_{i}$ indicates higher similarity:
  (6) $$C_{i}=\overset{n}{\underset{j=1}{\sum }}\omega_{j}\xi_{i}\left(j\right)\;i=1,2,\ldots m$$

Based on grey relational analysis and the five gait features, the assessment process consisted of two main parts as follows:

Part 1: Determination of the reference set *R*, minAbs and maxAbs based on the healthy subjects:
  (1) Four AGGs are selected from each healthy subject, the five gait features of each AGG are calculated, and a $X_{4m\times n}$ matrix is constructed (here *m* is the number of healthy subjects, and *n* is the number of features).
  (2) A reference matrix $X_{ref}$ is determined according to Lin et al.’s study [35] as Equation (7) shows
    (7) $$X_{ref}=\left(X_{ref}\left(1\right),X_{ref}\left(2\right),\ldots X_{ref}\left(n\right)\right)$$
    $$=\left(\frac{\sum_{i=1}^{4m}x_{i}\left(1\right)}{4m},\frac{\sum_{i=1}^{4m}x_{i}\left(2\right)}{4m},\ldots \frac{\sum_{i=1}^{4m}x_{i}\left(n\right)}{4m}\right)$$
  (3) A normalized matrix $Z_{4m\times n}$ is obtained by normalizing $X_{4m\times n}$ to positive indicators (the larger value is, the better) according to the comprehensive grey relational generating method mentioned in Chang et al.’s study [46], as shown in Equation (8).
    (8) $$Z_{i}\left(j\right)=\{\begin{array}{c} -\frac{x_{i}\left(j\right)}{X_{ref}\left(j\right)}+2\;\;x_{i}\left(j\right)>\;X_{ref}\left(j\right)\\ \;\frac{x_{i}\left(j\right)}{X_{ref}\left(j\right)}\;\;x_{i}\left(j\right)\leq \;X_{ref}\left(j\right) \end{array}$$
  (4) The reference set *R* and parameters minAbs and maxAbs are determined. Each element in the reference set *R* was determined as the optimal value of the five gait features of healthy subjects. In practice, *R* was set to the mean vector of the normalized matrix $Z_{4m\times n}$ as shown in Equation (9). Then minAbs and maxAbs were computed according to Equation (5):
    (9) $$R=\left(\frac{\sum_{i=1}^{4m}Z_{i}\left(1\right)}{4m},\frac{\sum_{i=1}^{4m}Z_{i}\left(2\right)}{4m},\ldots \frac{\sum_{i=1}^{4m}Z_{i}\left(n\right)}{4m}\right)$$
Part 2: Gait assessment of an unknown subject. When the basic parameters are obtained, the gait assessment of an unknown subject can be performed as follows:
  (1) Four AGGs of the subject are selected to construct the test set G(4×n).
  (2) A normalized matrix $\bar{G}$ is obtained by normalizing G according to (8).
  (3) The correlation coefficient $\xi_{i}\left(j\right)$ and correlation degree $C_{i}$ between $\bar{G}$ and *R* are computed by (4) and (5), respectively. In this case, the roles of five gait features were assumed to be equal, so the weight was set to 0.2.
  (4) The gait assessment score is calculated by Equation (10):
    (10) $$\mathrm{Score}=\overset{4}{\underset{i=1}{\sum }}C_{i}/4\times 100$$

### 2.5. Statistical Methods 

In this study, one-way ANOVA was used to examine the differences in P, V, N, H, S or scores among subjects in different groups, respectively. When groups exhibited unequal variances, an additional one-way-Robust test (Welch test) was conducted. If the test indicated a significant difference between group means, Tamhane’s T2 post hoc analysis was pursued for multiple comparisons of the means [47]. When the variances were not different between groups, Fisher’s least significant difference (LSD) post hoc analysis was used for multiple comparisons of the means. One-way repeated ANOVA was used to measure the differences between slow, normal and fast speed between subjects and LSD was used to multiple comparisons of the means. Reported results were considered significant for *p* < 0.05. Statistical analysis was performed using SPSS version 20.0 (SPSS Inc., Chicago, IL, USA).

## 3. Results

### 3.1. Validation of the Gait Segmentation Algorithm 

To verify the effectiveness of the gait segmentation algorithm based on peak detection of AP acceleration, a preliminary gait segmentation experiment was carried out firstly on five subjects including one healthy adult (AD9), one healthy child (TD5) and three CP children (CP8, CP12, CP22; selected from different GMFCS levels). The results are summarized in Table 2. In this experiment, the actual gait cycles detected by FSRs were used as references, and the validity of gait segmentation was described by sensitivity (the number of gait cycles correctly detected divided by the number of actual gait cycles) and positive predictive values (PPVs, the number of gait cycles correctly detected divided by the number of detected gait cycles) [48]. As shown in Table 2, two healthy subjects, AD9 and TD5, gave the best gait segmentation results. For the three CP children, although both sensitivity and PPV were lower than in the healthy subjects, the gait segmentation algorithm based on peak detection of AP acceleration also achieved an accurate gait segmentation. Specially, the PPV was approximately equal to the sensitivity for all three CP children, which meant that the number of actual gait cycles (detected by FSRs) was equal nearly to the number of detected gait cycles (detected by peak detection of AP acceleration).

### 3.2. Gait Acceleration Pattern Analysis Results of AD Subjects

During the data acquisition process, healthy adults walked at 0.93 ± 0.06 step/s and the correlation coefficients of the corresponding gaits acceleration signals among AD group are listed in Table 3. The high mean correlation coefficients (*r* ≥ 0.75) of SI-L, AP-L, SI-R, AP-R, SI-W, AP-W means that the acceleration signals of these six channels were relatively stable among healthy AD subjects and could represent their common walking characteristics, so the AGG and CGG extraction were conducted on these six channels in this study.

### 3.3. Characteristic Gait Graph (CGG)

**Figure 5:**
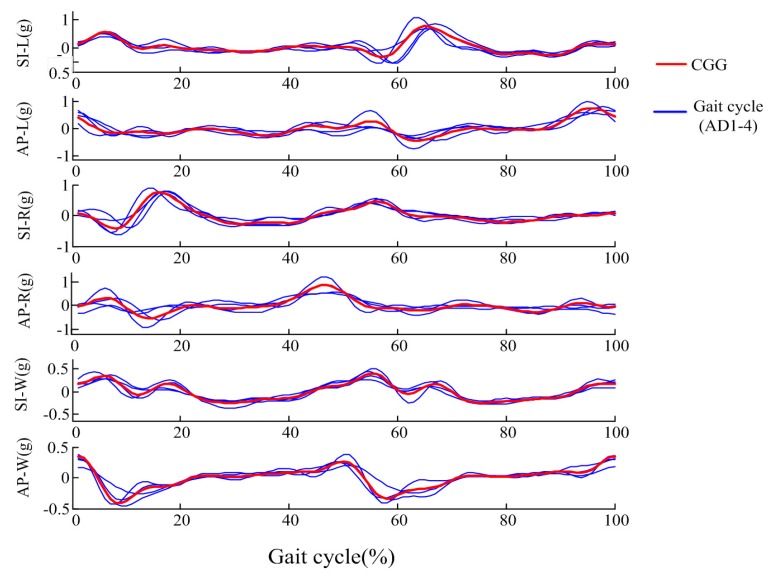
AGGs of AD1~AD4 and Characteristic Gait Graph (CGG).

In this study, the CGG was extracted as the average of AGGs of four healthy adults (AD1~AD4) to represent as standard gait acceleration pattern for further gait assessment. Figure 5 gives representative AGGs of AD1-4 and the extracted CGG. 

It can be observed that, although some differences exist in the AGGs, some basic acceleration pattern features could be observed in all subjects. As for CGG, in the subplot of AP-W, we can observe that: (1) the starting point with a high value corresponds to the time when the left foot touches the ground, and then the acceleration decreases quickly; (2) when the right foot starts to lift (at about 12% of the gait cycle), the signal began to rise; (3) the signal reaches a peak corresponding to the time when the right foot touches the ground (at about 50% of the gait cycle). The pattern from 0% to 50% of the stride cycle is similar to that from 50% to 100%. This phenomenon verifies the symmetry between left and right legs. Because of the support moment generated in the ankle, knee, and hip of the support leg, there are several peaks appearing in the 0–20%, 40–70% sections of the gait cycle in SI-W, SI-L, SI-R axes. In the SI-W graph, the acceleration signals in the 25–40% and 75–90% regions changed slowly, and the two largest peak points appeared at about 6% and 55%, corresponding to the foot flat according to Auvinet et al.’s study [49]. The curve in the 0–15%, 50–65% regions of the gait cycle in the SI-L axes was similar to the curve in the 50–65%, 0–15% part of the gait cycle in the SI-R axes, which reflects the symmetry of the left and right legs. For the AP-L axis, the acceleration presents a falling trend at first, and then rises. Similarly, the right foot swings firstly and stands later, so the AP-R acceleration presents a rising trend at first and begins to decrease at about 50% of the gait cycle.

### 3.4. Gait Acceleration Pattern Analysis of Different Groups

**Figure 6:**
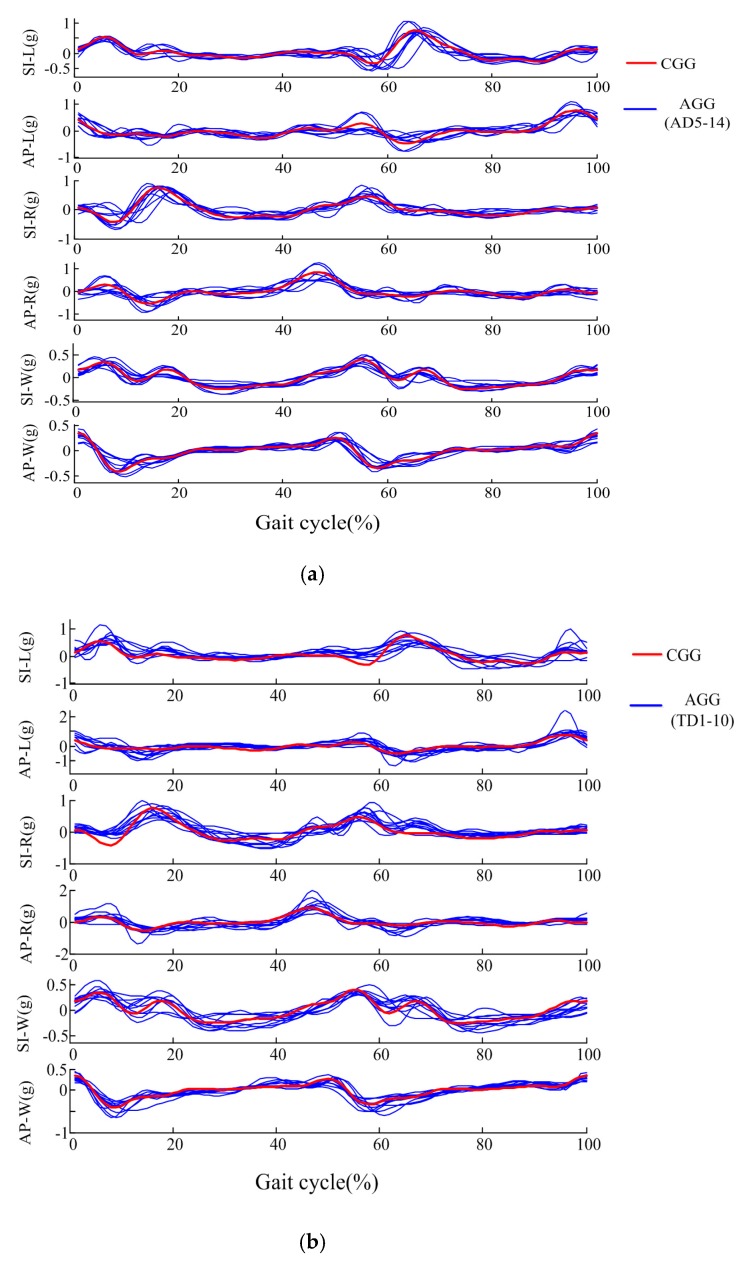

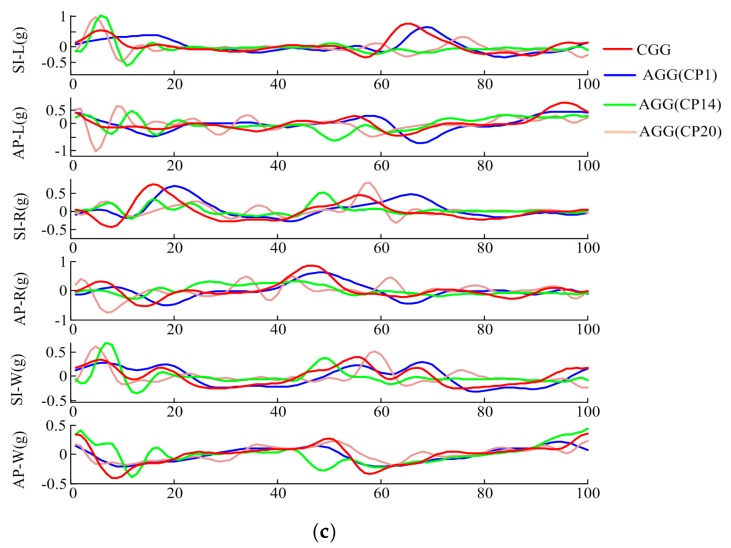
Comparisons between CGG and: (**a**) AGGs of AD group; (**b**) AGGs of TD group; (**c**) AGGs of three children with CP.

Figure 6 gives examples of the AGGs of the AD group (AD5~AD14), TD group and three children with CP. As seen in Figure 6a, the AGGs of healthy adults were similar to the CGGs, the correlation coefficient P between the AD group and CGG was 5.13 ± 0.45, and the correlation coefficient of AGGs among the AD subjects was 4.90 ± 0.56. For healthy children, as seen in Figure 6b, despite the age and gender differences of these healthy children, their gait patterns were also similar to the CGGs, the P between the TD group and CGG was 4.88 ± 0.43, and the correlation coefficient of AGGs among TD subjects was 4.59 ± 0.78. 

The gait patterns of the patients with CP were much more complex than those of healthy adults and healthy children. The correlation coefficient P between the CP group and CGG was 2.39 ± 1.33, and the correlation coefficient of AGGs among the CP group subjects was 2.14 ± 1.65. In Figure 6c, compared to CGG, the symptoms and severity of motor dysfunction could result in abnormality in the gait acceleration modes of CP patients. CP1 was classified as Grade I using GMFCS. Her lower limbs were almost normal and she could walk independently and continuously. We could see that the basic shape of CP1’s AGG was similar to the CGG, and most peak and valley points appeared in similar positions. CP14 was classified as hemiplegia left and Grade II using GMFCS. CP14’s right leg was almost normal, and she walked mainly depending on right leg and it was hard for her to bend her left leg. From the AGG of CP 14, it could be seen that some feature points disappeared compared to the CGG, especially in the SI-L and SI-W graph. In the AP-W graph, the peak at about 45% corresponding to the right foot touching ground was ahead of the CGG, which meant that the duration of the left step was short, and the value of this peak was much lower than the peak at 0%. CP20 had strong muscle strength compared to healthy subjects. She could not bend her legs easily, walked slowly, unstably, with the leg shaking, and was classified as Grade III using GMFCS. For SI-W axis, the signal in 15–55%, 65–100% changed slowly, which may be attributed to the fact that CP20 could not walk at a fast speed.

### 3.5. Gait Features Analysis of AGG

**Figure 7:**
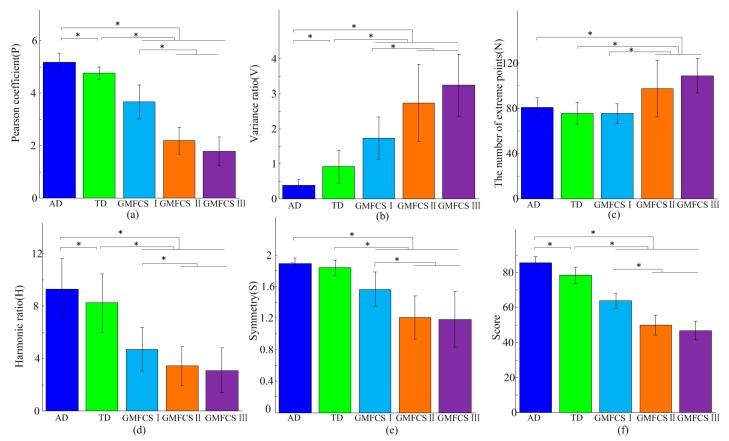
Five features and assessment scores of all subjects. (**a**) Pearson coefficient (P); (**b**) Variance ratio (VR); (**c**) The number of extreme points (N); (**d**) Harmonic ratio (HR); (**e**) Symmetry (S); (**f**) Assessment scores. “*” indicates statistically significant differences.

Figure 7a–e shows the features of the different groups, respectively. For the TD group, the values of N and S had no significant difference with the AD group (*p* = 0.142 and *p* = 0.057 respectively), however, there were significant differences in the other three parameters (P: *p* < 0.05; V: *p* < 0.05; H: *p* < 0.05). There was a certain regularity among CP children of different GMFCS levels. The values of P, H and S of CP showed a decreasing trend with the aggravation of symptoms of CP, and V and N presented an opposite trend. Significant differences existed in P, V, N, H, S between the AD group and subjects classified as GMFCS II, GMFCS III (*p* < 0.05 for all). Significant differences also existed in P, V, H, S (*p* < 0.05 for these four) between the AD group and CP with GMFCS I, but not for N (*p* = 0.091). Among the CP group, significant differences existed in P, N, H, V, S between GMFCS I subjects and GMFCS II subjects (*p* < 0.05 for all). However, there was no significant differences in P, V, N, H, S between subjects in the GMFCS II and GMFCS III groups (*p* = 0.095, 0.591, 0.479, 0.565 and 1.000, respectively).

### 3.6. Gait Assessment Results

**Figure 8:**
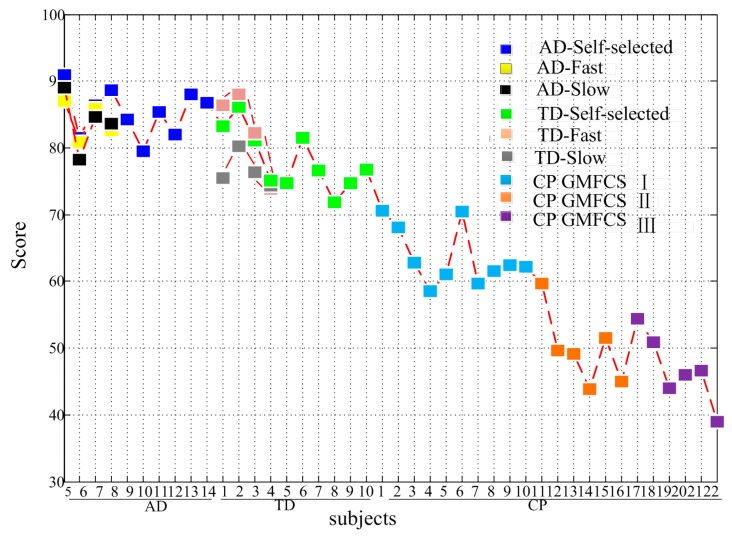
The gait assessment scores of all subjects.

In the establishment phase of the assessment model, data from four healthy subjects (AD1~AD4) were used to determine the parameters of the reference set *R*, minAbs and maxAb. Then, the gait of other subjects (AD5~AD14, TD1~TD10, CP1~CP22) were assessed according to the established model. As shown in Figure 7f, significant score differences existed between the AD group (Score = 85.33 ± 3.54) and the TD group (Score = 78.16 ± 4.56), CP in GMFCS I (Score = 63.73 ± 4.36), CP in GMFCS II (Score = 49.83 ± 5.66), and CP in GMFCS III (Score = 46.82 ± 5.35) (*p* ≤ 0.001 for all). Significant score differences also existed between the TD group and GMFCS I, GMFCS II, GMFCS III CP, respectively (*p* < 0.001 for all). Among the CP group, there were significant differences between GMFCS I and GMFCS II (*p* < 0.001), GMFCS I and GMFCS III (*p* < 0.001), but no significant differences between GMFCS II and GMFCS III (*p* = 0.260). Figure 8 gives the assessment results for each subject. For CP patients, the gait score differences within the same group were related to the patient’s dysfunction in some degree. For example, CP1, whose lower limb was almost normal, obtained the highest gait assessment score among GMFCS I. CP16 was an athetosic patient and more involuntary trembling appeared during his walking. CP16’s gait score was lower than other patients who were classified as GMFCS II. Among the patients in GMFCS III, the score of CP17 was obviously higher than others, and similar to the scores of subjects in GMFCS II. It may because CP17’s symmetry between the left and right legs was relatively normal. The quadriplegic ptient CP22 obtained the lowest gait assessment score.

To explore whether the walking speed impacted the assessment score, four healthy subjects (AD5~AD8) and four healthy children (TD1~TD4) were assessed at slow, self-selected and fast speeds (see Table 4) respectively. As shown in Figure 8, eight subjects obtained different gait assessment scores at low (Score = 80.25 ± 5.26), self-selected (Score = 84.11 ± 4.98) and fast (Score = 83.35 ± 4.62) speed. Significant differences existed between slow and self-selected speed (*p* < 0.05), fast speed (*p* < 0.05), but no significant differences existed between self-selected and fast speed (*p* = 0.497).

## 4. Discussion

### 4.1. Gait Acceleration Pattern of Different Groups

This study aimed to propose an acceleration-based method to assess the gait abnormality of cerebral palsy patients. AGG was defined to represent the gait pattern of each person. Being consistent with the study of Zijlstra et al. [22], high inter-subject correlation coefficients in the gait patterns were obtained in healthy adults. Similar gait patterns compared with healthy adults were also observed in healthy children. However, due to the immaturity and uncertainty of gaits, greater inter-subject variability existed in healthy children than in healthy adults during walking, as stated in Sutherland’s study [50]. Hence, in this study, the AD group was used to extract the standard gait pattern and calculate relative reference values. That was to say, as mentioned in the Methods part, the standard gait pattern CGG, the reference matrix $X_{ref}$ and reference set *R* used in the proposed gait assessment model were established based on healthy adults. For CP children, the AGG was more complex compared to healthy adults and healthy children. Various bone and muscle malformations during growth might affect the legs and the trunk and force children to adopt abnormal postures during walking [51]. 

### 4.2. The Effectiveness of Gait Features

In some related studies, some standard clinically relevant gait characteristics describing pace, rhythm, variability, asymmetry and postural control were often used to detect gait impairment [52,53]. Like these gait characteristics, the five gait features, including Pearson coefficient, variance ratio, the number of extreme points, harmonic ratio and symmetry, adopted in this study could also detect gait impairment in rhythm, variability and asymmetry, etc. The low variance ratio showed good repeatability in a number of gait cycles. In turn, a high variance ratio represented high gait variability. The harmonic ratio provided an indication of the smoothness and rhythm of gait patterns. The symmetry parameter could describe the similarity (low similarity means high asymmetry) of left and right legs during gait. In addition to the rhythm, variability and asymmetry, more gait abnormalities were considered in this study. For example, the Pearson coefficient (P) was proposed to depict the similarity between abnormal gait of CP subjects and the relatively standard gait of healthy subjects. Because children with CP, especially those with severe symptoms, often swung accompanied by some involuntary shaking during walking, the number of extreme points was used to detect gait stability. 

The results of this study confirmed that these gait parameters could be used to effectively detect gait impairment. The AD group and TD group obtained high symmetry values, which meant that gait symmetry was unaffected by age and consistent with Lythgo et al.’s study [54]. The gait repeatability of healthy children was lower than that of adults, which was consistent with the results that the stride-to-stride control of walking was not fully mature, even in 7-year-old children in the study of Hausdorff et al. [55]. Being consistent with the studies of Hausdorff et al. [55] and Iosa et al. [27], low repeatability, smoothness and symmetry were found to exist in the CP group. In the meanwhile, the gait features were found to be related to the degree of motor impairment of CP sufferers. For CP children with GMFCS I, whose symptoms were milder compared to those with GMFCS II and GMFCS III, the gait features were close to those of the healthy subjects. 

### 4.3. The Effectiveness of Gait Assessment Model

In comparison with the studies of Iosa et al. [27], the novelty of this study was that a gait assessment model based on the gait characteristics was proposed for the quantitative assessment of gait abnormalities of CP patients. The effectiveness of the proposed gait assessment model could be verified based on the following two aspects: firstly, the gait scores of the TD group were lower than those of the AD group. In particular, the effect of walking speed on the gait scores of healthy adults was less than on those of healthy children. Some recent studies have suggested that normalized basic gait parameters stabilize from 5 to 13 years, with little change from the age of seven [56], and it is not fully developed or mature by the age of 7 [57]. In this study, the children in the TD group were between 5 and 9 years old, it meant that some children may not have attained fully mature gaits, which may cause some differences in the kinematics and kinetics of gaits [57]. Secondly, the proposed gait assessment model could validly discriminate between healthy subjects and CP patients. The scores of healthy adults and children were significantly higher than those of CP children. More specifically, the experimental results demonstrated that the proposed assessment model could be used to evaluate gait abnormality in CP children.

It was worth mentioning that the data collection process should be standardized to ensure the effectiveness of the proposed gait assessment model. To obtain typical CGGs, healthy adults should walk at a normal manner, not deliberately swing around or shake up and down, and walk along a straight line. CP children could walk at their usual way, but their guardian should lead them to walk along a straight line to ensure the accuracy of the evaluation.

### 4.4. Limitations and Future Work

This paper reported a preliminary study on the evaluation of the gait motor dysfunction of CP children from the perspective of acceleration. Although some interesting results have been obtained, there were some limitations demanding further efforts. The main limitation of this study was that the gait of children with CP was mainly evaluated from a global perspective in this study, and more specific assessments of gait abnormality like reduced knee flexion, foot drop and the link to motor and structural deficits need a deeper exploration in future work. In addition, to further explore the influence of speed on this model, slow, self-selected and fast speeds of four healthy adults and four healthy children were analyzed. Two healthy children obtained lower gait scores at slow speed than self-selected and fast speeds. Based on the effect of walking speed on the assessment scores of healthy children, whether slow walking speed is one of the major factors resulting in CP children’s low gait scores need further exploration. 

## 5. Conclusions

In summary, this paper proposed an acceleration-based gait assessment method for children with cerebral palsy. By placing three IMUs on the waist and thigh to capture gait information during level walking, the differences in acceleration modes between children with CP and healthy subjects were compared, and five gait parameters, including Pearson coefficient, variance ratio, the number of extreme points, harmonic ratio and symmetry, were used to describe the differences. The gait assessment model was established based on grey relational analysis and these five gait parameters. The experimental results demonstrated that the proposed assessment model could effectively evaluate the abnormal degree of gait in CP children. This study provided an objective gait analysis method for CP sufferers that should prove useful in clinical diagnosis and rehabilitation treatment.

## Figures and Tables

**Table 1:** Information about the children with CP.

CP	Gender	Age (Years)	Type	GMFCS Level	Walking State	SF (Step/s)
1	F	12	Spastic, LN	I	WI	0.9
2	F	12	Spastic, HL	I	WI	0.94
3	M	8	Spastic, HR	I	WI	0.96
4	M	7	Spastic, HL	I	WI	0.97
5	F	7.6	Spastic, HR	I	WI	0.88
6	M	7	Spastic, HR	I	WI	0.96
7	M	7.2	Spastic, HL	I	WI	0.95
8	M	7.4	Spastic, HL	I	WI	0.98
9	F	3	Spastic, DI	I	WI	1.12
10	M	3	Spastic, DI	I	WI	1.01
11	M	8	Spastic, DI	II	WI	0.92
12	F	5	Spastic, DI	II	WI, SG	0.84
13	M	4	Spastic, DI	II	WI, SG	0.8
14	F	12	Spastic, HL	II	WI	0.72
15	M	9	Spastic, DI	II	WI	1.00
16	M	9	Athetosic, DI	II	WI	0.72
17	F	7.6	Spastic, DI	III	WI	0.72
18	F	6	Spastic, DI	III	WA	0.64
19	M	3.6	Spastic, QU	III	WA	0.62
20	F	7	Spastic, QU	III	WA	0.68
21	F	12	Spastic, DI	III	WI, SG	0.6
22	M	5	Spastic, QU	III	WA	0.67

GMFCS: scale of gross motor function classification system. A high GMFCS scale value means bad motor function. LN: the lower limbs are almost normal; HR: hemiplegia on the right limbs; HL: hemiplegia on the left limbs; QU: quadriplegia; DI: diplegia. WI: walking independently; WA: walking with assistance; SG: scissor gait; SF: stride frequency; For CP 19 and CP22, their guardians had to hold their arms from the side to prevent them from falling.

**Table 2:** Gait segmentation results of five subjects.

	AD9	TD5	CP8	CP13	CP22
**Sensitivity (%)**	100	96.67	86.67	96.67	83.34
**PPV (%)**	100	96.67	86.67	90.65	80.65

**Table 3:** Correlation coefficients of the 3 × 3 channels acceleration in the AD group.

Acceleration Channel	Mean.	S.D.
SI-L	0.79	0.12
ML-L	0.03	0.68
AP-L	0.78	0.10
SI-R	0.78	0.12
ML-R	0.05	0.70
AP-R	0.77	0.11
SI-W	0.89	0.05
ML-W	0.02	0.61
AP-W	0.88	0.06

**Table 4:** Speed information of eight healthy subjects.

Subjects	Slow	Self	Fast
	(Step/s)	(Step/s)	(Step/s)
AD5	0.78	0.85	0.96
AD6	0.76	0.88	0.93
AD7	0.83	0.96	1.1
AD8	0.89	1.01	1.13
TD1	0.82	0.94	1.04
TD2	0.90	1.05	1.17
TD3	0.95	1.10	1.14
TD4	0.85	0.93	0.99
